# Hypokalemic Paralysis due to Primary Sjögren Syndrome: Case Report and Review of the Literature

**DOI:** 10.1155/2017/7509238

**Published:** 2017-08-01

**Authors:** A. Garza-Alpirez, A. C. Arana-Guajardo, J. A. Esquivel-Valerio, M. A. Villarreal-Alarcón, D. A. Galarza-Delgado

**Affiliations:** Servicio de Reumatología, Departamento de Medicina Interna, Hospital Universitario “Dr. José Eleuterio González”, Universidad Autónoma de Nuevo León, Monterrey, NL, Mexico

## Abstract

Tubulointerstitial nephritis (TIN) is the main renal involvement associated with primary Sjögren syndrome (pSS). TIN can manifest as distal renal tubular acidosis (RTA), nephrogenic diabetes insipidus, proximal tubular dysfunction, and others. We present a 31-year-old female with hypokalemic paralysis due to distal RTA (dRTA). She received symptomatic treatment and hydroxychloroquine with a good response. There is insufficient information on whether to perform a kidney biopsy in these patients or not. The evidence suggests that there is an inflammatory background and therefore a potential serious affection to these patients, such as hypokalemic paralysis. We found 52 cases of hypokalemic paralysis due to dRTA in pSS patients. The majority of those patients were treated only with symptomatic medication. Patients who received corticosteroids had stable evolution even though they did not have another symptomatology. With such heterogeneous information, prospective studies are needed to assess the value of adding corticosteroids as a standardized treatment of this manifestation.

## 1. Introduction

Sjögren's syndrome is an autoimmune disease with glandular (salivary and lacrimal) and extraglandular (neurologic, renal, hepatic, respiratory, vascular, and cutaneous) manifestations. Tubulointerstitial nephritis (TIN) is the main renal involvement associated with primary Sjögren syndrome (pSS). TIN can manifest as distal renal tubular acidosis (RTA), nephrogenic diabetes insipidus, proximal tubular dysfunction, and others [1], of which RTA is the main clinical presentation [2]. RTA has been reported in 4.3 to 9% of pSS patients; it is more common in middle-aged women, and two-thirds of them will develop symptoms [2, 3]. Hypokalemic paralysis is the initial symptom in seven percent of patients with Sjögren's syndrome [4]. We present a case of paralysis due to RTA in a pSS patient and also discuss the treatment in these patients.

## 2. Case Report

**Figure 1:**
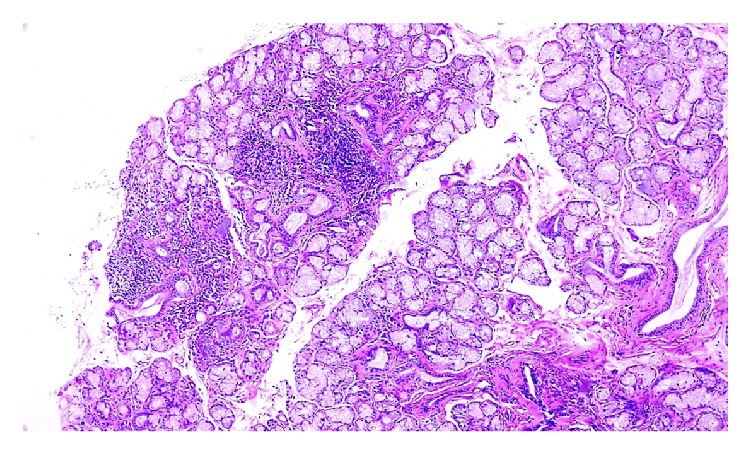
A panoramic photo of minor salivary gland biopsy. A chronic lymphocyte focal sialadenitis was observed.

A 31-year-old female presented to the emergency room due to a 3-day history of progressive weakness and pain of the upper and lower extremities until walking was impossible. Two days before admission, cramps and generalized dysesthesias were evidenced. On admission, the patient presented mild dyspnea. Her past medical record was significant for polyarthralgias in carpal, metacarpophalangeal, and proximal interphalangeal joints and dry mouth for the past three months. She denied use of alcohol, illicit drugs, or herbal medicines. Her vital signs on admission were a temperature of 36.3°C, a heart rate of 54 beats per minute, a respiratory rate of 20 breaths per minute, oxygen saturation of 97% at room air, capillary blood glucose of 103 g/dL, and blood pressure of 100/60 mmHg. On physical examination, the deep tendon reflexes were globally diminished, her muscle strength, both proximal and distal, was 3/5 on Lovett's scale, and her tongue was dry and the infralingual salivary pooling was absent. Remarkable laboratory tests are shown in Table 1. A panoramic photo of minor salivary gland biopsy is shown in Figure 1. With all lab results, a distal RTA (dRTA) diagnosis due to pSS was made. Hypokalemia and metabolic acidosis were treated with intravenous potassium chloride and sodium bicarbonate. Then, we initiated hydroxychloroquine. The patient was discharged and we followed her up in our clinic every two months for the next eight months. She was reported to be asymptomatic with the use of potassium citrate only.

## 3. Discussion

A recent set of classification criteria for pSS were published by the ACR/EULAR in 2016 [6] and this applies to the individual that has a score of ≥4. According to this, the diagnosis of this autoimmune disease was made in our patient (labial salivary gland with a focus score of ≥1, anti-SSA positive, and an unstimulated whole saliva flow of less than 0.1 mL/min). Renal involvement in pSS is the result of two distinct pathophysiological processes: TIN and glomerulopathy [1]. The tubulointerstitial inflammation is the most common renal lesion described by Talal et al. [7]. dRTA prevalence fluctuates between 5 and 70%, according to population studies [4, 8, 9]. dRTA can be classified as complete or incomplete; the former is characterized by metabolic acidosis with morning urine pH > 5.5 and a positive urinary anion gap. The incomplete form presents with normal serum bicarbonate levels but urinary pH fails to fall to <5.3 after ammonium chloride loading [10]. The pathogenic mechanism of this complication is not completely understood. Antibodies to vacuolar H+-ATPase and anion exchanger 1, as well as antibodies to carbonic anhydrase II, have been implicated in the pathogenesis [11–13]. Another hypothesis is a defective S-phase-kinase-associated protein-1, a component of the regulator of the ATPase of vacuolar and endosomal membranes that could induce a defective V-ATPase assembly [14]. Also, a possible relation between antibodies anti-SSA/Ro and dRTA has been described as one pathogenic mechanism of development [15].

Hypokalemia is the most common electrolyte abnormality in patients with dRTA. The causes of hypokalemia include decreased distal tubular Na delivery, secondary hyperaldosteronism, defective H-KATPase, and bicarbonaturia [16]. Hypokalemic paralysis may precede sicca syndrome from three months to four years in patients with a final diagnosis of pSS [17, 18].

Renal biopsy is not mandatory in these patients [2], but it may help us evidence the inflammatory mechanisms that trigger the disease. As has been demonstrated by Evans et al. in twelve patients with TIN secondary to pSS, they observed CD4+ T-cell predominance in biopsies, similar to those seen in lip salivary glands [19]. Also, similar lymphocytic infiltrates around renal tubules have been observed [20]. More data from prospective studies of pSS biopsies are needed in order to enhance knowledge in these subsets of patients and also to determine the best treatment.

dRTA treatment includes potassium restitution before alkali therapy, because the last might aggravate hypokalemia by enhancing the shift of potassium into cells and bicarbonaturia [21]. In the beginning, hydroxychloroquine was started in the suspicion of a secondary cause of Sjögren's syndrome but it was later discontinued.

RTA is not a usual indication for immunomodulatory therapy in pSS, even though it is an extraglandular manifestation [22]. Steroid therapy in cases that are nonresponsive to replacement therapy and in those with recurring hypokalemic paralysis attacks is indicated [23].

We searched in MEDLINE, IMBIOMED, and Google Scholar for clinical cases of hypokalemic paralysis due to pSS. We included only articles written in English or Spanish. In Table 2, we describe each one of them: number of cases, age of patients, extraglandular manifestations besides dRTA, treatment, and outcome. We found fifty-two cases for analysis but we included only cases with a complete report of treatment [15, 21–47]. We observed the highest frequency of this clinical presentation in young adults of the female gender. It is important to note that, in some cases, dRTA was present before the diagnosis of pSS. All patients received symptomatic treatment. We noted that 25% (13/52) received corticosteroids. Of these patients, 61% (8/13) did not report extraglandular manifestations, besides dRTA. The outcomes (at different duration) were clinically stable in 61% (8/13), 8% (1/13) had a relapse after treatment was stopped, 8% (1/13) died from an infectious cause, and 23% (3/13) did not report the outcome.

On the other hand, 32.6% (17/52) received only symptomatic treatment. Of these patients, 41% (7/17) did not report extraglandular manifestations. Only in six (35%) patients was the outcome reported, of whom 83% (5/6) were clinically stable, and in 17% (1/6) four relapses occurred.

In the early diagnosis era of autoimmune diseases (like in rheumatoid arthritis), the importance of recognizing kidney involvement before glandular symptoms appear has been observed previously [21, 25, 28]. Also, we consider it important to determine whether some factors can trigger the beginning of this manifestation. This association has been observed by Logan and Ahmed. They described in a patient the use of* Echinacea* as a trigger of pSS [45]. Perhaps this means that the immunological tolerance is already lost, and some infections or substances can precipitate the clinical disease. We agree with the recommendation given by François and Mariette to screen all pSS patients according to manifestations every six to twelve months [10].

With such heterogeneous information, prospective studies are needed to assess the value of adding corticosteroids as a standardized treatment of this manifestation. We may consider that, in cases of hypokalemic paralysis in which there is a potentially life-threatening presentation, the treatment with corticosteroids could be justified.

## Figures and Tables

**Table 1:** Laboratory investigation.

Laboratory investigation	Result
CBC	Hemoglobin: 14.7 g/dL, WBC: 8.7 × 10^3^, lymphocytes: 0.683 × 10^3^, platelets: 159 K/*μ*L
Serum electrolytes	Sodium: 138.2 mmol/L, potassium: 2.7 mmol/L, chloride: 101 mmol/L
Serum chemistry	Glucose: 123 mg/dL, creatinine: 0.8 mg/dL, urea nitrogen: 13 mg/dL
Liver panel	AST: 19 IU/L, ALT: 13 IU/L, albumin: 4.2 g/dL, total bilirubin: 0.7 mg/dL
Urinalysis	pH: 8, leucocytes: 0–2/HPF, erythrocytes: 0/HPF, tubular cells: 0/HPF
Urinary electrolytes	Sodium: 114 mmol/L, potassium: 32 mmol/L, chloride: 57.3 mmol/L, creatinine: 31.8 mg/dL
Urinary anion gap	76 mmol/L
Blood gas	pH: 7.12, HCO_3_: 11 mmol/L, pO_2_: 31 mmHg, pCO_2_: 37 mmHg, saturation: 37%
Serum anion gap	10 mEq/L
Thyroid panel	TSH: 2.06 *μ*IU/mL, free T4: 0.94 ng/dL
Acute phase reactants	ESR: 31 mm/h, CRP < 0.5 mg/L
Virus panel	HIV-negative, HBV-negative, HCV-negative
Rheumatoid factor	IgM: 155.7 IU/mL, IgG: 6.7 IU/mL, IgA: 12.2 IU/mL
ANAs by IFA	1 : 5120 fine speckled
SSA/SSB by ELISA	200.14/19.67 IU/mL
Unstimulated whole saliva flow, without anesthesia	1.4 mL/15 minutes
Minor salivary gland biopsy^*∗*^	Positive, focus score of 5
Schirmer's test	Right eye: 7 mm, left eye: 10 mm

^*∗*^According to [5].

**Table 2:** Comparative treatment in hypokalemic paralysis due to distal RTA in Sjögren syndrome patients.

Reference	Type of study	Number of patients	Age (years) mean	Extraglandular manifestations besides dRTA	Treatment	Follow-up	Outcome
Goroshi et al.	Case series	13	33.1	Arthritis, arthralgias, vasculitis	Symptomatic Extraglandular: HCQ and MTX	2.8 years (0.5–4)	No improvement in reduction of HCO_3_ or K requirements

Khadgawat et al.	Report of cases	2	20.5	No	Symptomatic	Not reported	—

Soy et al.	Case report	1	39	Arthralgia, myalgia, nephrolithiasis	Symptomatic and methylprednisolone	2 years	Stable clinical evolution

Cheng et al.	Report of cases	2	76	Nephrocalcinosis	Symptomatic and prednisolone	5–12 months	Stable clinical evolution

Kawashima et al.	Case report	1	39	Osteomalacia, interstitial nephritis	Symptomatic and prednisolone	2 years	Relapse after stopping treatment

Comer et al.	Case report	1	43	No	Symptomatic	2 years	Stable clinical evolution

Seirafian et al.	Case report	1	64	No	Symptomatic, prednisolone, and HCQ	Not reported	—

Vaidya and Ganeshpure	Case report	1	23	No	Symptomatic	1.5 years	Stable clinical evolution

Sarah et al.	Report of cases	2	35	No	Symptomatic	Not reported	—

Rao et al.	Report of cases	3	37	Not reported	Symptomatic	Not reported	—

Nail et al.	Case report	1	65	No	Symptomatic	Not reported	—

Rajagopala et al.	Case report	1	36	Medullary nephrocalcinosis, recurrent CNS demyelination, neuromyelitis optica Secondary APS with thrombosis	Symptomatic, methylprednisolone, prednisolone, CYC, and AZA	3 months	Stable clinical evolution

Palkar et al.	Case report	1	58	Low-grade fever	Symptomatic	Not reported	Stable clinical evolution

Dasari et al.	Case report	1	40	No	Symptomatic and prednisolone	6 months	Stable clinical evolution

Singhvi et al.	Case report	1	30	No	Symptomatic and prednisolone	6 months	Stable clinical evolution

Chang et al.	Report of cases	2	10	One patient: carotid artery stenosis	Symptomatic	3–6 years	One patient: four relapses

Eriksson et al.	Report of cases	6	64.6	Not reported	No reported	Not reported	—

Taylor and Parsons	Case report	1	55	No	Symptomatic	Not reported	—

Carminati et al.	Case report	1	32	No	Symptomatic	Not reported	—

Muthukrishnan et al.	Case report	1	39	No	Symptomatic and prednisolone	2 years	Stable clinical evolution

Prakash et al.	Case report	1	49	No	Symptomatic, methylprednisolone, prednisolone	16 days	Died

Skalova et al.	Case report	1	16	No	Symptomatic, methylprednisolone, CYL	Not reported	Stable clinical evolution

Liao et al.	Case report	1	49	Not reported	Symptomatic	Not reported	—

Sengul et al.	Case report	1	48	No	Symptomatic, prednisolone, HCQ	Not reported	—

Yilmaz et al.	Case report	1	53	No	Symptomatic, methylprednisolone, HCQ, AZA	10 days	Stable clinical evolution

Logan and Ahmed	Case report	1	36	No	Symptomatic, HCQ	3 years	Stable clinical evolution

Fujimoto et al.	Case report	1	27	Kidney lithiasis	Symptomatic	4 months	Stable clinical evolution

Mugundhan et al.	Case report	1	38	Nephrocalcinosis	Symptomatic and prednisolone	Not reported	—

Garza-Alpirez et al.	Case report	1	31	Polyarthralgias	Symptomatic, HCQ	8 months	Stable clinical evolution

Symptomatic: potassium (K) and bicarbonate (HCO_3_); HCQ: hydroxychloroquine; MTX: methotrexate; CYC: cyclophosphamide; AZA: azathioprine; MM: mycophenolate mofetil; CYL: cyclosporine A; APS: antiphospholipid syndrome; extraglandular manifestation: arthritis, arthralgia, and vasculitis.
